# Presence of a Physician Safety Champion Is Associated with a Reduction in Urinary Catheter Utilization in the Pediatric Intensive Care Unit

**DOI:** 10.1371/journal.pone.0144222

**Published:** 2015-12-14

**Authors:** Samara Zavalkoff, Nadine Korah, Caroline Quach

**Affiliations:** 1 Division of Critical Care, Department of Pediatrics, The Montreal Children’s Hospital, McGill University Health Centre, Montreal, QC, Canada; 2 Division of General Pediatrics, Department of Pediatrics, The Montreal Children’s Hospital, McGill University Health Centre, Montreal, QC, Canada; 3 Infection Control, Department of Pediatrics, The Montreal Children’s Hospital, McGill University Health Centre, Montreal, QC, Canada; 4 Division of Infectious Diseases, Department of Pediatrics, McGill University, Montreal, QC, Canada; 5 Department of Epidemiology, Biostatistics and Occupational Health, McGill University, Montreal, QC, Canada; University of Milan, ITALY

## Abstract

**Background:**

Safety champions are effective in a variety of safety initiatives; however, there are no reports of their role in hospital-acquired infections prevention.

**Objective:**

We aimed to describe the association of the presence of a physician safety champion with our urinary catheter device utilization ratios (DUR) in the Pediatric Intensive Care Unit (PICU).

**Methods:**

Our PICU has incidence rates of catheter-associated urinary tract infections (CAUTI) and urinary catheter DUR above the 90^th^ percentile. Using a quasi-experimental design, we compared our DUR when the PICU team was exposed and unexposed (champion’s maternity leaves) to a physician safety champion. Hospital acquired infection (HAI) surveillance of all PICU admissions between April 1^st^ 2009 and June 29^th^ 2013 was done prospectively. To ensure stable acuity of the patient population over time, we used the central venous catheter (CVC) DUR as a control.

**Results:**

The urinary catheter DUR was 0.44 (95% confidence interval [CI] 0.42–0.45) during the unexposed period versus 0.39 (95%CI 0.38–0.40) during the exposed period, for an absolute difference of 0.05 (95%CI 0.03–0.06; p<0.0001). The overall CVC DUR increased from 0.57 (95%CI 0.55–0.58) during the unexposed period to 0.63 (95%CI 0.61–0.64) during the exposed period, an absolute increase of 0.06 (95%CI 0.04–0.08; p<0.0001). Comparing the exposed and unexposed periods, adjusting for time trend, we observed a 17% decrease in the urinary catheter DUR when the safety champion was present (odds ratio [OR] 0.83; 95%CI 0.77–0.90). The rate of catheter-associated urinary tract infections did not change.

**Conclusions:**

The presence of a unit-based safety champion can have a positive impact on urinary catheter DUR in a PICU.

## Introduction

Clinical champions have been promoted as a key element of successful patient safety initiatives[1–4]. They often emerge informally with strong enthusiasm for a safety project [5]. A champion’s main objective is to convince team members to adopt a practice change using education and advocacy[2]. To be successful, the champion also needs to build positive relationships with the involved end users and know how to navigate barriers specific to their setting[2].

The effectiveness of safety champions has been demonstrated in a variety of safety initiatives such as increasing reporting of adverse events, implementation of rapid response teams and implementation of computerized physician order entry systems[2,6,7]. To our knowledge, there is no report of the role of a safety champion towards preventing hospital-acquired infections (HAI) in an intensive care unit although their use has been recommended[8]. HAIs remain a significant cause for morbidity and mortality in healthcare, affecting 1.7 million people a year, with 99, 000 associated deaths, in the United States alone[9]. Specifically, our targeted infection, catheter-associated urinary tract infections (CAUTI), has an associated mortality of 2.3% across all age groups[9].

Our Pediatric Intensive Care Unit (PICU) has had incidence rates of CAUTI above the 90^th^ percentile for many years when compared to other PICUs participating in the National Healthcare Safety Network, with a urinary catheter device utilization ratio (DUR) that is also above the 90^th^ percentile consistently since at least 2009[10]. Although several efforts and strategies were used to decrease urinary catheter utilization in our PICU, we only started to see a change in our DUR when a physician champion, dedicated to patient safety, was hired. These prior strategies included education and advocacy from the infection control team and an interdisciplinary informal agreement by the PICU team to round on the presence and need for urinary catheters.

The objective of this study is to describe, through a quasi-experimental design, the association between the presence of a physician champion and the urinary catheter DUR in our PICU of a tertiary-care hospital.

## Methods

Informed consent was not requested, as this study was a secondary analysis of data that is collected routinely for infection control monitoring. IRB approval was not sought for the same reason. One of the authors (SZ) is an attending physician in the PICU and did interact with patients who had urinary catheters; however, she was not involved in data collection or analysis. The administrative agent doing data entry anonymized the data upon entry into the database. The author who analysed the data (CQ) only had access to anonymized data with unique ID numbers

### Study setting and population

The Montreal Children’s Hospital of the McGill University Health Centre is a tertiary-care pediatric hospital. Its 12-bed PICU is a mixed medical, surgical and cardiac unit with approximately 730 annual admissions, for an average of 3,628 annual patient-days during the study period. We performed a secondary data analysis of our HAI surveillance database, using an ecological study design that included all children admitted to the PICU between April 1, 2009 and June 29, 2013. IRB approval was not sought as this secondary analysis was performed on data previously collected for infection control monitoring. During the PICU study period, the only change to the attending group and schedule was the hire of one part time intensivist in January 2012. There were no changes to the PICU population makeup, but an increasing trend in total admission and patient days over the course of the study.

### Infection control/Patient safety program

In April 2010, the safety champion was hired as a clinician and a quality improvement officer, as other clinicians are hired to be educators or clinician-scientists. This additional leadership role is not accompanied by any additional income. Given our high rates of CAUTI and urinary catheter DUR, she elected to target this particular device. The champion’s first step was to increase awareness of the problem: it was repeatedly highlighted by the champion at the PICU’s monthly interdisciplinary quality improvement rounds, reported on in the PICU quality improvement newsletter, discussed in bedside rounds and run charts of urinary catheter DUR and CAUTI were displayed in the PICU. The champion led the creation of an interdisciplinary policy for the on service team to systematically round on the presence and possibility of removal of the urinary catheter in each patient on a daily basis. This policy was formally disseminated though interdisciplinary quality improvement meeting minutes, the quality improvement newsletter and the nursing communication book, but also informally by the champion through discussion with team members when on and off the unit. At quarterly quality improvement *follow-up* rounds, the champion sought feedback from the group on adherence, successes and barriers to upholding the policy. In addition, while on service (12 weeks/year) in the PICU, she educated team members about the problem and advocated for catheter removal, when medically appropriate. During the study period, there was no other formal safety champion, from any discipline; however, informal, self-appointed champions of the project, impassioned by the physician champion, were not measured. The PICU team did not implement a bundle to reduce the use of urinary catheters of CAUTI, at any point, during the study period.

During the study period, urinary catheters were inserted and removed when clinically indicated as per the treating team; there were no formal criteria for insertion or removal. A central venous catheter (CVC) insertion bundle and a ventilator associated pneumonia preventive bundle were implemented in 2010 (year 2 of study). Although the champion supported these projects, her main focus was the use of urinary catheters given the specific high rates of urinary catheter use and CAUTI infections in the study’s PICU. In comparison, the PICU’s rate of catheter associated blood stream infections was approximately at the 25^th^ percentile compared to National Healthcare Safety Network[10]. In addition, although not measured, there was a greater buy-in from the interdisciplinary PICU team to reduce urinary catheter use with a sense that there was overuse in the PICU. In contrast, the team felt that the use of CVCs was very judicious and would be difficult to reduce further.

### Study design and outcome

We used a quasi-experimental design to compare urinary catheter DUR when the PICU team was exposed and unexposed to the safety champion. Periods of non-exposure occurred during the champion’s maternity leaves. To ensure that the acuity of the patient population did not change over time, we used CVC DUR as a control device, not targeted by the champion or other team members. The exposed and unexposed periods are detailed in Table 1.

**Table 1:** Physician Champion: PICU exposed and unexposed periods.

Start date	End date	Exposed	Comments
April 1^st^ 2009	March 21^st^, 2010	NO	Before physician champion’s arrival
April 1^st^ 2010	July 31^st^ 2010	YES	
August 1^st^ 2010	January 31^st^ 2011	NO	Maternity leave
February 1^st^ 2011	June 18th 2012	YES	
June 19^th^ 2012	February 28^th^ 2013	NO	Maternity leave
March 1^st^ 2013	June 29^th^ 2013	YES	

PICU: Pediatric Intensive Care Unit

### HAI surveillance, definitions and outcomes

Our prospective HAI surveillance program has existed since 1985 and has been described previously [11]. In summary, our surveillance year starts on April 1 and ends on March 31 of the next year and is composed of thirteen 28-day periods. Our definitions for UTI and CAUTI have remained unchanged since 2009. HAI surveillance nurses review laboratory data and medical records and complete a standardized case report form to establish UTI occurrence. They also document the presence of a urinary catheter or CVC for each patient on a daily basis. The infection control practitioner and the infection control physician adjudicate cases, based on information collected. We use the American National Healthcare Safety Network definition for CAUTI[12–15].

The number of patient-days was defined as the total number of days that patients spent in the PICU. The number of urinary catheter-days was defined as the total number of days the patient was exposed to catheter. In the case of CVC days, if a patient had more than one central catheter on a given day, this counted as one catheter-day.

### Statistical analysis

We calculated a DUR by dividing the total number of catheter-days (urinary or CVC) by the total number of patient-days. Odds ratios (OR) were calculated to compare DUR during exposed and unexposed periods. Using a binomial regression (PROC GENMOD, binomial distribution, canonical link), we compared exposed and unexposed periods for urinary and CVC DURs, adjusting for year. Statistical significance was determined using 2-sided *p-*values (p<0.05). All statistical analyses were done using SAS 9.2 (SAS Institute, Cary, NC).

## Results

During the study period (4.25 years), approximately 3110 children were admitted to the PICU. The median number of children with a urinary catheter varied from five to six per day. Fifty-two CAUTIs occurred during the study period, for a CAUTI incidence rate that varied from a low of 6.6 (2011–2012) to a peak of 11.5 (2010–2011) per 1000 urinary catheter-days. There was no significant change in our CAUTI rate throughout the study period. During the same period, 21 central line associated bloodstream infections occurred with an incidence rate that varied from 1.61 (2010–2011) to 3.43 (2011–2012) per 1000 CVC-days. Table 2 describes the study population stratified by year.

**Table 2:** Characteristics of the study population.

Study year*	Patient-days (n)	Median daily number of patients with devices (IQR 25–75)			Device-days (DUR)	
		Urinary catheters	CVC	ETT	Urinary catheters	CVC
2009–10	3102	4 (3–5)	5 (4–7)	4 (3–5)	1379 (0.44)	1885 (0.64)
2010–11	2971	4 (2–5)	5 (5–5)	4 (3–5)	1390 (0.47)	1823 (0.61)
2011–12	3244	3 (2–4)	5 (4–6)	3 (2–5)	1059 (0.33)	1747 (0.54)
2012–13	3223	3 (2–4)	5 (4–6)	4 (3–5)	1264 (0.38)	1853 (0.56)
2013–14**	766	3 (2–4)	6 (5–7)	4 (3–6)	260 (0.34)	766 (0.73)

* Study year: from April 1^st^ to March 31^st^ of subsequent year

** Partial year: periods 1–3, inclusively

PICU: Pediatric Intensive Care Unit; DUR: Device utilization ratio; CVC: Central venous catheter; IQR: Interquartile range

**Fig 1:**
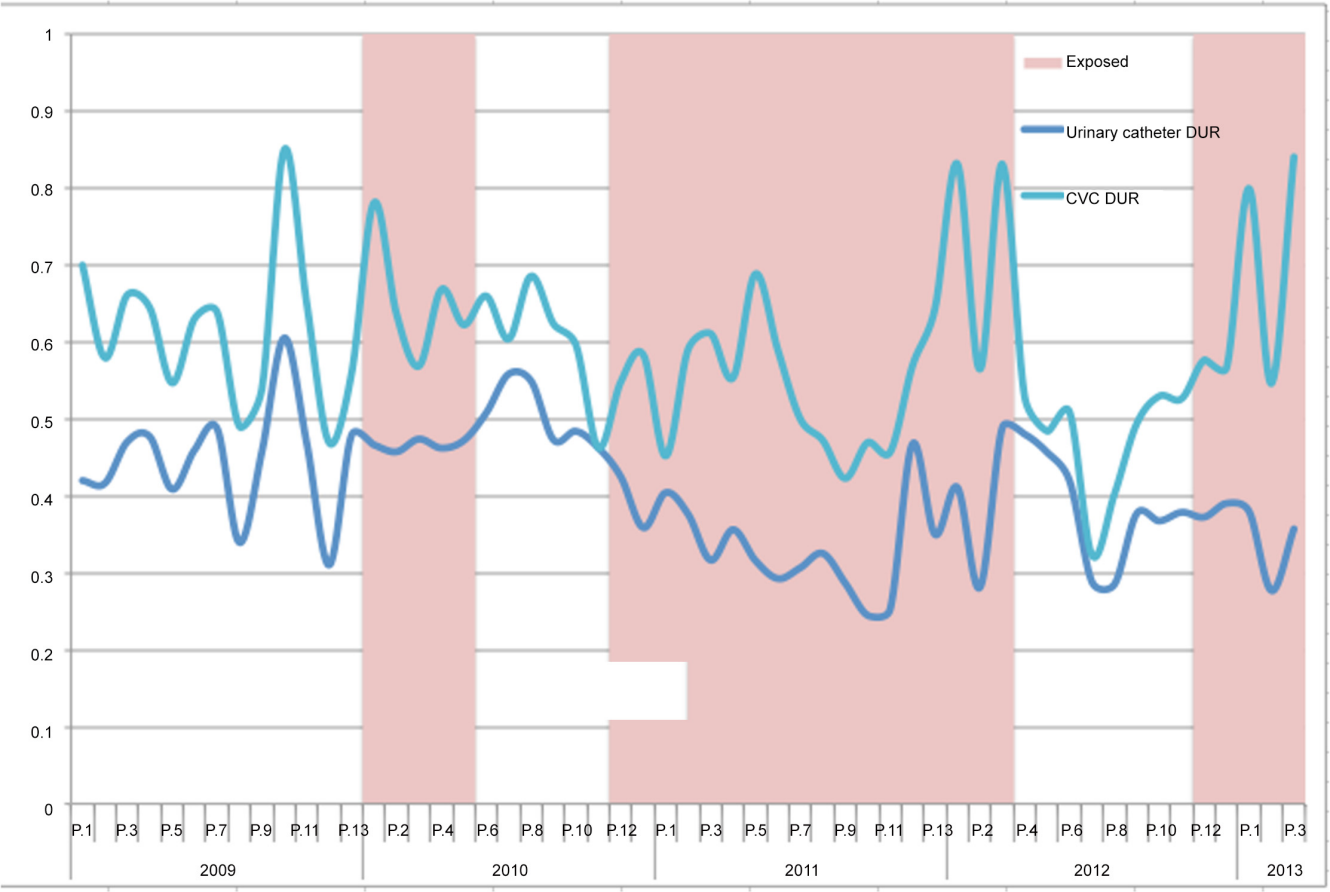
Device utilization ratio (urinary catheter and CVC) by period and exposure status.

Table 3 details DUR for both urinary catheters and CVC in the PICU when exposed and unexposed to the physician champion. Fig 1 illustrates DUR for urinary catheters and CVC by period and exposure status. The overall DUR for urinary catheters was 0.44 (95% confidence interval [CI] 0.42–0.45) during the unexposed period compared to 0.39 (95%CI 0.38–0.40) during the exposed period, for an absolute difference of 0.05 (95%CI 0.03–0.06; p<0.0001) and a crude relative risk (RR) of 0.89 (95%CI 0.86–0.93). The overall CVC DUR increased from 0.57 (95%CI 0.55–0.58) during the unexposed period to 0.63 (95%CI 0.61–0.64) during the exposed period, an absolute increase of 0.06 (95%CI 0.04–0.08; p<0.0001) and a crude RR of 1.10 (95%CI 1.07–1.14).

**Table 3:** Device Utilization Ratio (device-days/patient days) per Exposure Group and Study Year.

Study Year*	Urinary catheter DUR		CVC DUR	
	Exposed	Unexposed	Exposed	Unexposed
2009–2010	-	0.44 (1379/3102)	-	0.61 (1885/3102)
2010–2011	0.52 (864/1654)	0.50 (663/1317)	0.72 (1189/1654)	0.60 (794/1317)
2011–2012	0.33 (1059/3244)	-	0.54 (1747/3244)	-
2012–2013	0.38 (513/1357)	0.38 (751/1972)	0.67 (915/1357)	0.48 (938/1972)
2013–2014**	0.34 (260/766)	-	0.73 (559/766)	-
**Pooled**	**0.38**	**0.44**	**0.63**	**0.57**

* Study year: April 1^st^ until March 31^st^ of subsequent year

** Partial year: periods 1–3, inclusively

DUR: Device utilization ratio; CVC: Central venous catheter

Data were analyzed using binomial regression. Comparing the exposed and unexposed periods, adjusting for time trend, we observed a 17% decrease in the catheter DUR when the safety champion was present (odds ratio [OR] 0.83; 95%CI 0.77–0.90). By opposition, we observed a 22% increase in the CVC DUR when comparing the exposed and unexposed periods (OR 1.22; 95% CI 1.13–1.31).

## Discussion

The presence of a physician, whose role is to champion patient safety in the PICU, was associated with a decrease in urinary catheter utilization, a device that our organization targeted given our high rates of use compared to benchmarks. Comparing exposed to randomly selected non-exposed periods (maternity leaves), in a quasi-experimental design, we showed a 17% decrease in urinary catheter DUR. A stable CVC DUR, during the study period, supports that the decrease in urinary catheter DUR was not due to a decrease in PICU patients’ severity of illness. This is corroborated by an increased percentage of patients requiring mechanical ventilation in the PICU from 49% in 2010 to 54% in 2011 to 58% in 2012 (local data).

With the involvement of champions, other studies have shown improvements in the implementation of a wide range of health care change initiatives[2,6,7,16–18]. Conversely, not having any champion for a change process can be detrimental[19]. Like in our study, these champions were described to play important roles in education and advocacy.

Champions, also known as opinion leaders in contexts outside of medicine, are described to be effective by using several strategies. These include bridging knowledge and practice, bringing enthusiasm and laying a path for acceptance of an innovation [2,20] -all tasks our safety champion carried out. A local champion, like ours, may be successful in these tasks as he or she understands the context where the change initiative will occur. This is critical, as innovation success can be highly context-specific [5]. In addition, a champion helps establish a safety culture that is essential to any success safety improvement project[3,6].

Like other work describing the role of a champion in the implementation of a change initiative [16], we failed to demonstrate an improvement in patient outcome–in our case CAUTI rates. Campbell et al demonstrated that the introduction of “nurse champions significantly improved compliance with ICU sepsis screening (…), but had virtually no effect on patient outcomes related to percentage for patients treated for sepsis[16].” This supports the notion that safety initiatives are most successful when they are bundled actions[21–24]. Similar evidence exists for change initiates outside of healthcare that promote multiple levels of influence to create a sustainable change[25]. In essence, a champion may be necessary, but not sufficient for a change initiative to impact patient outcomes.

This notion is further supported by the lack of sustainability of our reduction in catheter DUR during our champion’s absences from the PICU. Sustainability of any change is said to be the greatest challenge in safety improvement[26]. Further work on this project will strive to complete the transition from an individual champion-led strategy to a unit-based culture change. This may require the involvement of other types of champions (e.g. executive or managerial) who can “leverage their respective organizational positions and networks to forward [an] implementation process[2].”

Our study has some limitations. First, this study is a retrospective analysis of data and is therefore subject to the quality of data previously collected. Our HAI surveillance data and denominators are, however, collected using standardized definitions and case report forms and have not changed during the study period. The infection control team is stable; the same surveillance nurses have been working in our division for more than seven years. Moreover, as this is a real-life study, it is impossible to have an exact measure of the exposure. One can only say when the champion was present and when she wasn’t. The intensity of her impact on the PICU team is impossible to quantify. Yet, we have seen a difference in the urinary catheter DUR that is not explained by time trend alone; a decrease that is not seen in the CVC DUR. Lastly, we do not have demographic and severity of illness data to ensure homogeneity of the study population observed in the two periods. Nevertheless, the mandate of the PICU did not change throughout the study period, no new programs were taken on or lost and admission/discharge criteria were stable, so we expect the population was homogenous.

In conclusion, our work demonstrates that a local, clinical patient safety champion can help to reduce urinary catheter utilization in a PICU. Our study also furthers understanding of the roles such a champion may take on in a safety initiative, yet highlights the importance of a multi-pronged approach to achieve sustainable change.
